# Graphene-Based Polymer Composites for Flexible Electronic Applications

**DOI:** 10.3390/mi13071123

**Published:** 2022-07-16

**Authors:** Ana M. Díez-Pascual, Abbas Rahdar

**Affiliations:** 1 Universidad de Alcalá, Facultad de Ciencias, Departamento de Química Analítica, Química Física e Ingeniería Química, Ctra. Madrid-Barcelona, Km. 33.6, 28805 Alcalá de Henares, Madrid, Spain; 2 Department of Physics, University of Zabol, Zabol P.O. Box 98613-35856, Iran; a.rahdar@uoz.ac.ir

**Keywords:** graphene, polymer composites, flexible electronics, transistors

## Abstract

Graphene-based nanomaterials have gained a lot of interest over the last years in flexible electronics due to their exceptional electrical, mechanical, and optoelectronic properties, as well as their potential of surface modification. Their flexibility and processability make them suitable for electronic devices that require bending, folding, and stretching, which cannot be fulfilled by conventional electronics. These nanomaterials can be assembled with various types of organic materials, including polymers, and biomolecules, to generate a variety of nanocomposites with greater stretchability and healability, higher stiffness, electrical conductivity, and exceptional thermal stability for flexible lighting and display technologies. This article summarizes the main characteristics and synthesis methods of graphene, its oxidized form graphene oxide (GO), and reduced GO derivative, as well as their corresponding polymeric composites, and provides a brief overview about some recent examples of these nanocomposites in flexible electronic applications, including electrodes for solar cells and supercapacitors, electronic textiles, and transistors.

## 1. Introduction

**Figure 1:**
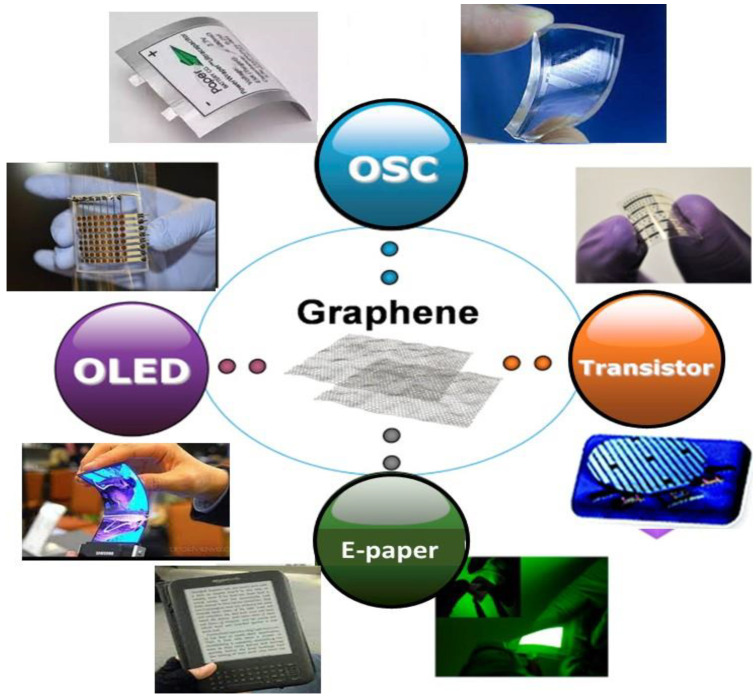
Representation of the applications of graphene in flexible electronics.

Graphene is a 2D multifunctional carbon nanomaterial of a few nanometer thickness, comprising sp^2^ carbon atoms forming a layered structure. It is considered as the starting point of other carbon nanomaterials with different dimensionalities, such as 0D fullerenes, 1D CNTs, and 3D graphite, since these can be assembled based on this unit [1]. In 2004, it was exfoliated for the first time from graphite at Manchester University by Geim and Novoselov [2], and displays exceptional physical and electronic properties, very high mobility of electrons, around 3 × 10^5^ cm^2^/(V·s), and therefore superior electrical conductivity [3]. It is one of the lighter materials on earth, and it is believed to be the stiffest, with an intrinsic strength close to 130 GPa [4], a fracture toughness of 4 MPa, fracture stress of 98 GPa, and modulus of elasticity close to 1100 GPa [5], being much stronger than steel. It has a thermal conductivity of around 5000 W/(m·K) [6], greater than that of Cu, and a huge surface area of 2600 m^2^/g. It can behave as a metal and semiconductor, but has no bandgap, therefore it is regarded as a semi-metal [7]. Furthermore, it is optically transparent, though its transmittance decreases with increasing number of graphene layers. Graphene monolayer has a transmittance of ∼97.8% [8], which is a key benefit for use in optoelectronic devices. In addition, it is biocompatible, and has good electrochemical stability. The combination of mechanical flexibility, its good electrical properties and huge surface area make this nanomaterial perfect for application in many fields, including flexible energy storage devices, such as lithium-ion batteries and supercapacitors, as well as wearable and portable electronics, such as touch screen displays, electronic papers, foldable organic light-emitting diodes (OLEDs), field-effect transistors (FETs), and so forth (Figure 1). 

**Figure 2:**
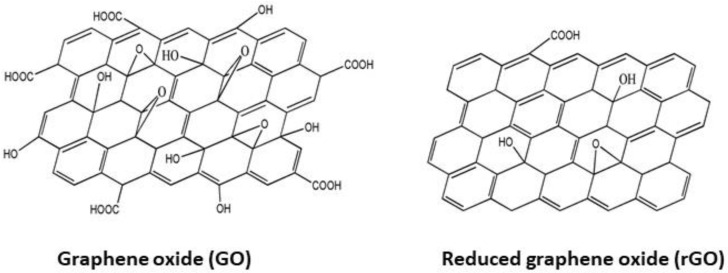
Representation of graphene derivatives: GO and rGO.

Nonetheless, some issues need to be solved before using graphene, including the fact that it is hydrophobic, hence insoluble in water, which strongly limits some applications. A way to solve this and extend its potential uses is to synthesize graphene derivatives, like graphene oxide, an oxidized form of graphene with carboxyl, epoxy, and hydroxyl groups on the planes of graphite (Figure 2). These oxygenated groups make GO amphiphilic, hence can be dispersed in many conventional solvents and can be processed in aqueous solution [9]. This good processability enables to develop thin films on many substrates through conventional drop-casting route, making it an appropriate candidate for flexible applications. In addition, the oxygenated moieties act as reactive points for GO modification via covalent and non-covalent approaches [10,11,12]. Moreover, GO films are optically transparent, non-toxic, and biocompatible. Nonetheless, this nanomaterial has considerably lower electron mobility than pristine graphene, and its electrical mobility is very low, hence it is electrically insulator. It also has poorer mechanical strength and thermal stability. 

The chemical or thermal reduction in GO can reestablish its conductivity to some degree. The resulting reduced graphene oxide (rGO) supports certain functional groups (Figure 2), which enables good dispersability in numerous solvents. In addition, it is fairly easy to regulate its solubility and electrical performance by adjusting the amount of residual functional groups [13]. The properties of rGO are midway between those of pristine graphene and GO. Accordingly, rGO has an electrical conductivity in the range of 10^2^–10^4^ times lower than raw graphene.

Over recent years, multifunctional wearable and flexible electronics have received a lot of interest. Remarkable efforts have been recently focused on developing multifunctional materials with an inherent flexible or stretchable property, such as waterproof wearable sensors with good mechanical durability and long-term stability [14] 

**Figure 3:**
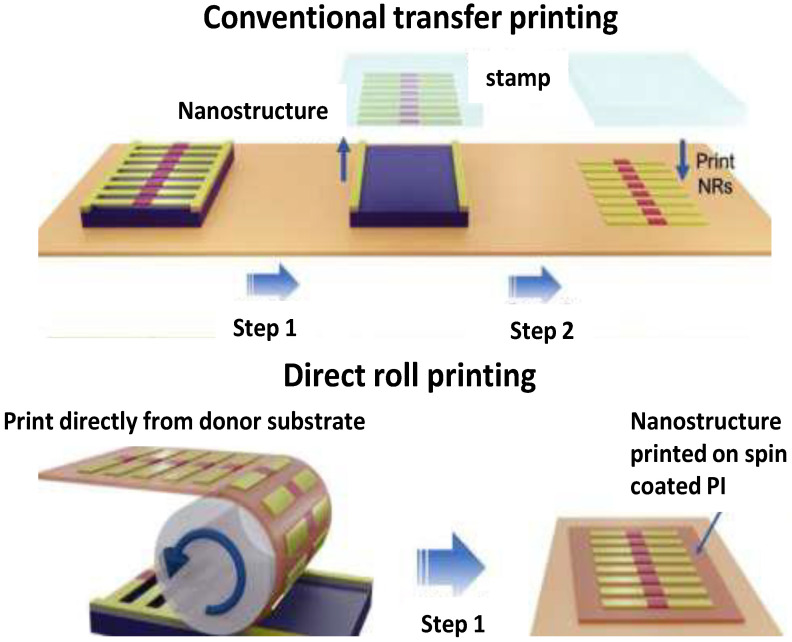
Representation of the most common technologies for the preparation of flexible electronics: conventional transfer printing and direct roll printing. Adapted from ref. [15,17].

Up to now, flexible electronics have been primarily manufactured by a three-step wet transfer printing process [15] (Figure 3). First, a silicon-based semiconductor nanostructure is grown on a substrate. Secondly, the nanostructure is taken from the substrate by a polymeric stamp. Finally, the nanostructure is transferred from the stamp to another flexible substrate. However, this process poses several constraints that make it challenging for large-scale applications, since the precise control of the transfer velocity, nanostructure adhesion, and orientation are difficult to attain reproducibly. Despite novel methods being developed to make the transfer more effective, like dry transfer printing, they often require additional equipment, such as lasers, thus increasing manufacture cost. An instant dry transfer printing technology has been reported by Heo et al. [16], based on the fact that materials expand at different rates when heated. Placing the device to be printed onto the surface to be anchored and then increasing the temperature, cracks are formed between the layers, allowing them to be detached successfully after printing. This novel dry transfer printing method is faster than the wet approaches and preserves the device initial shape and structure.

Zumeit et al. [17] have developed an alternative approach named “direct roll transfer”. Firstly, a thin silicon nanostructure is prepared. Then, the polymeric substrate (polyimide) is covered by a thin layer of chemicals and wrapped around a metal tube. A computer-controlled machine rolls the tube over the silicon wafer, transferring it to the flexible material. By thoughtfully optimizing the process, homogenous prints up to 96% transfer yield have been developed. Moreover, the manufacture of conductive materials in a quick, economic, and sustainable way is one of the main prerequisites for flexible electronics [15]. In this regard, a simple, fast, and sustainable flexible electronics preparation technology was reported by Wang et al. [18] to address the key restraints of materials and fabrication techniques. They prepared a thermoplastic polyurethane (TPU) membrane by electrospinning, which was used as substrate in a sandwich structure, assembled layer by layer, and each layer was composed of a TPU membrane, and a liquid metal printed on it. This strategy provides flexible devices, such as circuits, resistors, capacitors, inductors, and others, with excellent stretchability, air permeability, and stability. More importantly, they are reconfigurable, and address the concerns regarding environmental and energetic problems, opening new possibilities for commercialization. 

Nonetheless, despite the above-mentioned efforts, there is a lack of review articles summarizing the recent progress in this brilliant area. This article reviews the synthesis of graphene and its derivatives, along with its corresponding polymeric nanocomposites and offers a brief overview about some current examples of these nanocomposites in flexible and wearable electronics, including organic solar cells (OSC), supercapacitors, electronic textiles, and field-effect transistors (FETs). 

## 2. Synthesis of Graphene and Its Derivatives

### 2.1. Synthesis of Graphene

**Figure 4:**
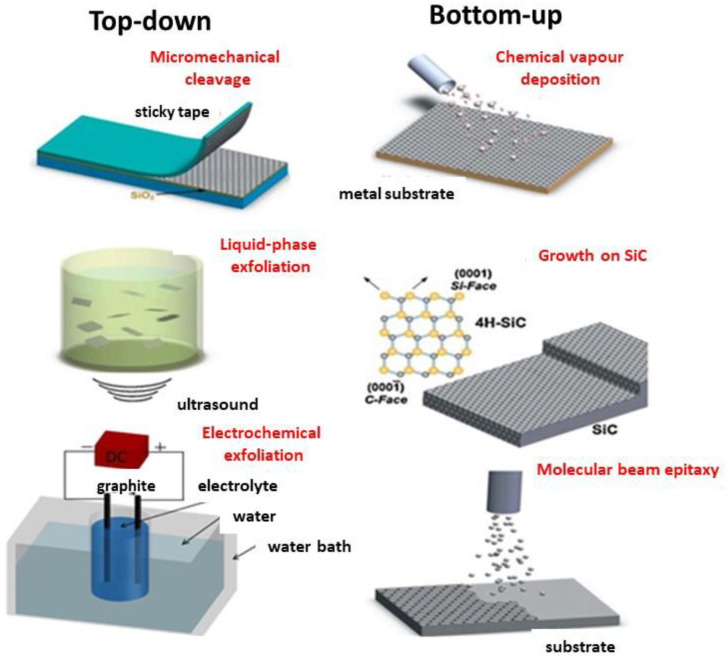
Top–down and bottom–up approaches for graphene manufacture.

The first attempt to synthesize graphene was reported by Boehm et al. [19] in 1961, who prepared extremely thin carbon lamellae by deflagration of graphite oxide via heating or reduction in alkaline medium. Geim and Novoselov [2] prepared graphene by peeling a graphite surface with scotch tape in 2004, and were awarded the Noble Prize in 2010. This approach yields high-quality monolayer graphene and it is economical, however it produces very low amounts and therefore it can only be used at lab level. Until now, synthesis can be accomplished through top–down and bottom–up procedures, as shown in Figure 4. Graphite can be exfoliated in liquid media, both in aqueous and non-aqueous solvents, via application of ultrasounds, in a process known as liquid-phase exfoliation (LPE) [20]. This is the key technique for manufacturing large amounts of high quality and low cost 2D materials, and it is now broadly accepted by both academia and industries since it is suitable for large mass production. It is typically performed in three steps: firstly, sonication causes the breakage of bulky flakes and the formation of twist band striations. Secondly, cracks develop along these striations, resulting in the unzipping of thin graphite layers upon intercalation of solvent. Thirdly, the thin layers are exfoliated into graphene. This method holds potential for application in optoelectronics and nanocomposites. Another means of exfoliation is the electrochemical method, in which ions enter within the graphite flakes and induce layer separation [20,21,22]. Thus, an applied voltage drives ionic species to intercalate into graphite where they form gaseous species that expand and exfoliate individual graphene sheets. The characteristics of the obtained graphene depend on the voltage and the electrolyte nature. This technique is cheap and sustainable, and could also be suitable for electronic applications. However, this approach is not a suitable manufacturing route due to several issues: only graphite monoliths are appropriate as a source for electrochemical exfoliation. In addition, due to the degradation of the graphite rod, the yield is too low and needs additional removal of unexfoliated material.

Regarding the bottom–up approaches [20], these begin with small molecular precursors as units using procedures, such as chemical vapor deposition (CVD), epitaxial growth, or molecular beam epitaxy (Figure 4). CVD is a method in which carrier gases and carbon precursors are inserted into a chamber at elevated temperature. The precursor is disintegrated to yield graphene on metal catalyst, such as Cu, Pd, Ru, or Ni [21]. It takes place in two steps: (1) the decomposition of precursors on the substrate surface at elevated temperatures with the aid of metals; then (2) the growth of graphene from the detached C atoms. This method is appropriate for use in flexible electronics. The key disadvantages are the potential presence of impurities to form the catalyst, the difficulty to tailor the film thickness, and the expensiveness of the substrate. 

Epitaxial growth is usually achieved on a SiC substrate in which graphite is decomposed by heating. This procedure allows to adjust the thickness by controlling temperature and time, and results in high-quality big layers with uniform thickness. It allows the direct production of electronic devices. 

### 2.2. Synthesis of Graphene Oxide

**Figure 5:**
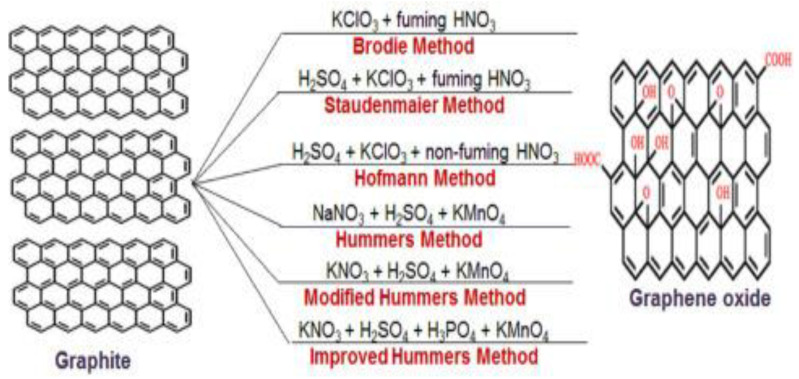
Graphene oxide synthesis by chemical oxidation using Brodie’s, Staudenmaier’s, Hofmann, and Hummer’s methods [27].

Different means to synthesize GO from graphite have been published including Brodie, Staudenmaier, Hofmann, and Hummers (Figure 5). Graphite oxide was first produced by Brodie in 1859 using KClO_3_ and HNO_3_ [23]. Then, Staudenmaier [24] and Hofmann used concentrated H_2_SO_4_, KClO_3_, and HNO_3_ to synthesize oxidized graphite. Based on these works, Hummers and Offeman in 1958 developed a novel path by replacing HNO_3_ and KClO_3_ with NaNO_3_ and KmnO_4_ [25], which has been the most used since 2004, when graphene was prepared for the first time. Nonetheless, it still has several drawbacks, including poor yield and toxic gas generation. In addition, there is high oxidant consumption, and it takes a long time, resulting in expensiveness and poor scalability. Thus, numerous works have been published to improve this synthesis. Environmentally friendly means that syntheses with natural oxidants like citric acid have also been reported [26], which circumvented the production of poisonous gases, appropriate for energy storage applications.

### 2.3. Synthesis of Reduced Graphene Oxide

Reduced graphene oxide (rGO) can be obtained from GO via removal of some oxygenated groups by chemical, thermal, and other methods [28]. The goal is to attain nanomaterials comparable to raw graphene. Nonetheless, due to the generation of defects and the existence of residual functional groups, the properties of rGO are midway between those of GO and graphene.

One approach is to heat GO at elevated temperature under inert, vacuum, or reducing atmosphere. However, this route is difficult to be applied for layers deposited onto substrates, consequently it is not suitable for electronic applications. Additional paths are irradiation with microwaves or reduction with a pulsed laser or an arc-discharge lamp [29], which results in extremely conductive rGO layers, allowing straightforward manufacturing of flexible devices. 

**Figure 6:**
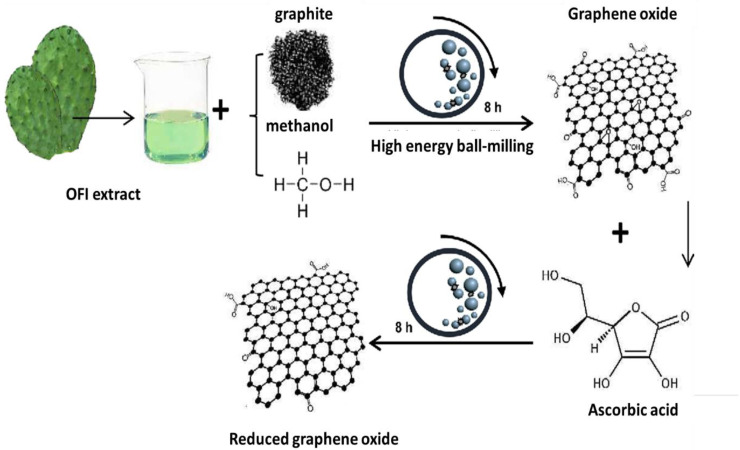
Sustainable production of rGO via high-energy ball milling using the reducing agent Opuntia ficus-indica (OFI) plant extract. Reproduced from [33], copyright 2017, Springer.

On the other hand, chemical reduction can be carried out at ambient conditions or with moderate heating using strong reductors, such as hydrazine hydrate [30]. Since this reagent is very toxic, alternative chemicals, such as Fe, hydroiodic acid (HI), sodium borohydride (NaBH_4_), hydroquinone, and hexamethylenetetramine [31,32] can be used. In addition, the global sustainability concern has motivated researchers to examine the application of bio-reducers derived from plants, bacteria, fungi, and so forth. For instance, a strong reducing agent can be derived from the Opuntia ficus-indica (OFI) plant [33] (Figure 6), which, combined with high-energy ball milling, can lead to sustainable and cheap, few layered rGO at an industrial level. rGO is well suitable for various applications, such as field effect transistors (FET), transparent conductors, and solar cells [34].

## 3. Graphene-Based Polymeric Composites for Flexible Electronics

**Figure 7:**
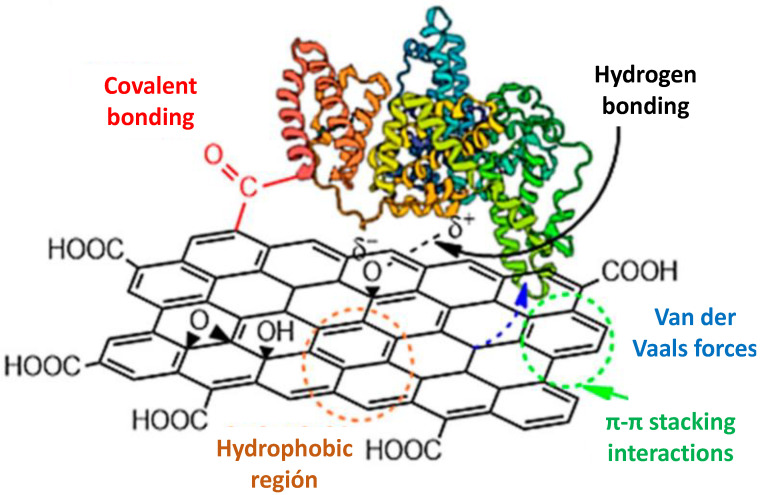
Non-covalent interactions between graphene and polymers. Adapted from [36].

The first work on polymer/graphene nanocomposite was published by Stankovich et al. [35] in 2006 via exfoliation of graphene with polystyrene as polymer host. Two types of interactions can occur between graphene related nanomaterials and polymer matrices, namely covalent and non-covalent. In the case of covalent functionalization, covalent bonds are formed between the polymer and the nanomaterial. However, for non-covalent strategies, many interactions can take place, including hydrophobic, π–π, van der Waals, ion–π, hydrogen bonding, and electrostatic (Figure 7) [36]. Among these, the weakest forces, van der Waals interactions, affect all neighboring atoms. The hydrophobic effects are a main contribution to consider in G and GO systems. π–π interactions occur in systems with aromatic rings. In addition, there is also a small chance that CH···π-like interactions happen around the edge of graphene that may be terminated with a hydrogen atom or from phenyl rings oriented perpendicular to the graphene surface. Since graphene is several magnitudes larger than the contributing part of the polymer, there could be numerous interactions on both sides of the nanomaterial sheet. On the other hand, the electrostatic interactions are more pronounced on GO, since the different oxygenated functional groups can be deprotonated depending on the environment. In addition, H bonding interactions are quite common between GO and polymers incorporating amine or hydroxyl groups, such as polyester amide copolymers, poly(vinyl alcohol), polyols, etc. 

**Figure 8:**
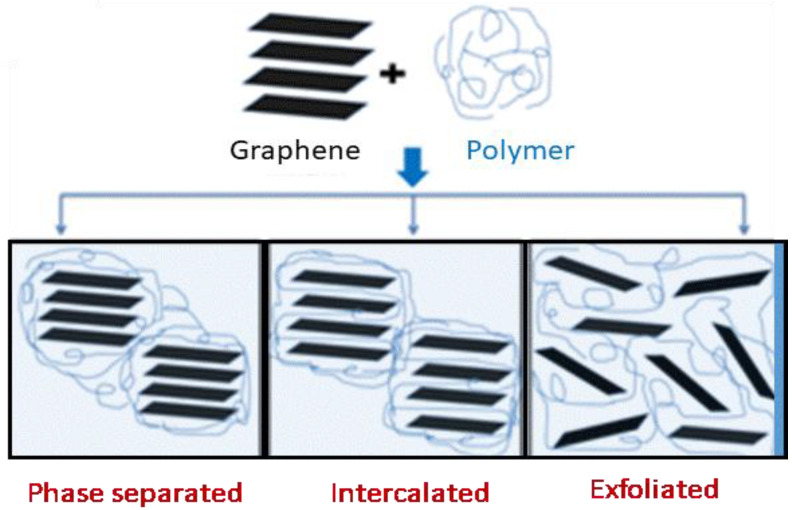
Schematic illustration of different types of graphene–polymer nanocomposites.

Taking into account these interactions, three different structures can be observed in graphene/polymer nanocomposites: phase separated, intercalated, and exfoliated (Figure 8). Intercalation is the desirable to occur, in which the polymer inserts into the spaces between the nanomaterial sheets. However, exfoliation is the most favorable, since the polymer is completely distributed within the individual nanometer layers.

The outstanding properties of graphene/polymer composites [37] arising from the above-mentioned types of interactions, grant their applications in flexible electronics, that is, electronics with performance equivalent to that of conventional technologies founded on rigid systems, through bendable and flexible arrangements. It can also imply lower cost and electronic system integration via using scalable engineering procedures such as printed electronics, roll-to-roll or lamination, which are not available for traditional materials. The following sections describe representative examples of graphene-based polymer nanocomposites used in flexible electronic devices.

### 3.1. Graphene-Based Electrodes in Solar Cells

Flexible electrodes based on graphene and its derivatives have a lot of potential in energy storage. For instance, a flexible nanocomposite consisting of poly(3,4-ethylenedioxythiophene) (PEDOT)–graphene was fabricated by electrochemical deposition of ethyl glycol on a graphene-filtrated carbon cloth substrate [38]. This flexible composite showed excellent capacitive properties. Poly(3,4-ethylenedioxythiophene)-poly(styrene sulfonate) (PEDOT:PSS) has also been mixed with graphene oxide derivatives via solution processing [39], yielding nanocomposites with improved properties for organic solar cells (OSC) applications [40]. Thus, these nanomaterials have been used as transparent conducting electrodes to substitute usual ITO electrodes in polymer solar cells. Numerous studies have dealt with flexible polymers, such as polyethylene terephthalate (PET) as substrate. Thus, thermally annealed rGO films were deposited onto PET and plasma treated to attain a hydrophilic surface. The OSC manufactured by spin coating [41] with rGO films 16 nm thick showed the highest efficiency (0.8%) and a transmittance of 65%. In addition, the device could resist more than 1300 cycles without lessening performance, whilst traditional ITO-based cells tend to break after a thousand of cycles, due to the fragility of ITO. Better performance was obtained by depositing a laser-patterned rGO micromesh on to PET, attributed to the mesh greater transmittance and reduced resistance compared to rGO sheets [42]. The main drawback of these type of electrodes is their high defect content that restraint device efficacy.

Flexible transparent electrodes based on sulfonated graphene/PEDOT have also been fabricated via in situ polymerization of the monomer EDOT using NaBH_4_ as reducing agent. The resulting nanocomposite was easily processable in water and organic solvents, and had elevated conductivity, stability, and transmittance [43]. Other electrodes have been prepared via spin coating a mixture of surfactant-functionalized graphene and PEDOT:PSS [44], and the conductivity and transparency were comparable to those of ITO electrode together with higher mechanical stability. Though, from an application viewpoint, the surfactant is unwanted.

**Figure 9:**
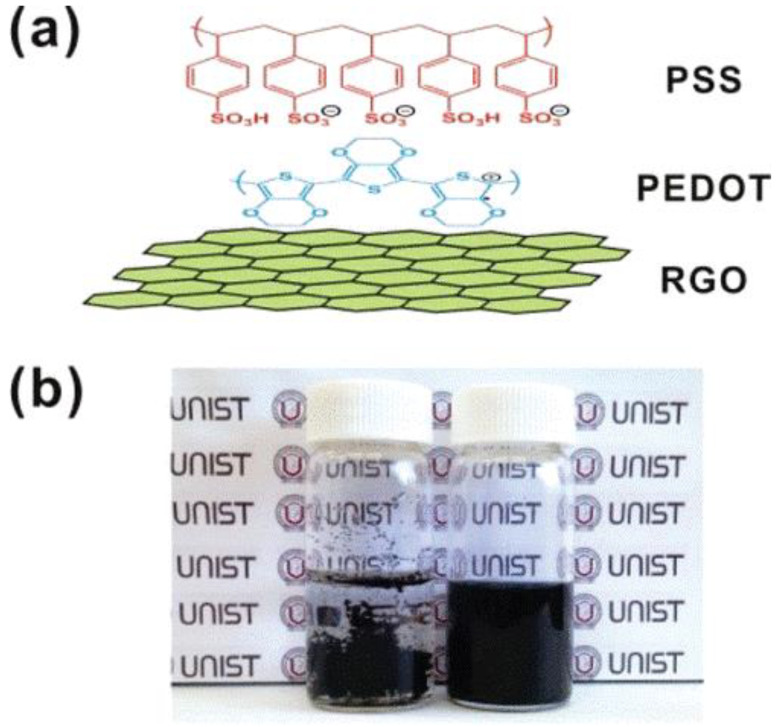
(**a**) Schematic representation of reduced graphene oxide (RGO) nanosheet with PEDOT:PSS. (**b**) Image of thin film of RGO/PEDOT deposited on a flexible PET substrate with a transmittance of 90%. Reproduced from [45], copyright 2011, with permission from the American Chemical Society.

An aqueous G dispersion in PEDOT:PSS was produced by a reduction in GO with this polymer, without the requirement for surfactants. This tactic comprises strong rGO-PEDOT π–π interactions between rGO sheets and the PEDOT chains, and intermolecular repulsions between PSS chains holding negative charges and covalently anchored to the rGO sheets. The film displayed a high conductivity combined with a transmittance of 90% (Figure 9) [45]. Other authors [46] fabricated flexible OSC on polyimide (PI) substrates with multilayer CVD graphene/PEDOT:PSS nanocomposites and gold nanoparticles as a top transparent electrode. The device kept a maximum efficiency of ~3.2% after a thousand cycles, representative of superior elasticity and durability. More significantly, it was reported that air did not penetrate the graphene layers, thus providing an outstanding packaging. Multilayer graphene can behave as a barrier against air pollution, which makes the device manufacture simpler and diminishes the related expenses.

Exfoliated graphene have also been deposited on flexible poly(ethylene 2,6-naphthalate) (PEN) substrates, and the resulting nanocomposite was used as an anode in OSCs. This anode showed a high transmittance, a small sheet resistance, an efficiency of 4.2% and a high stiffness that were maintained after 150 bending cycles. Flexible OSCs incorporating a low-pressure CVD graphene with a PEDOT:poly(ethylene glycol) (PEDOT:PEG) block copolymer were developed. They had very good conductivity and transparency and were applied as anode and cathode in conventional and inverted cells [47], reaching efficiencies of 6% and 7%, respectively. These G-based devices did not lose mechanical performance after hundreds of flexing cycles.

### 3.2. Graphene in Flexible Capacitors

**Figure 10:**
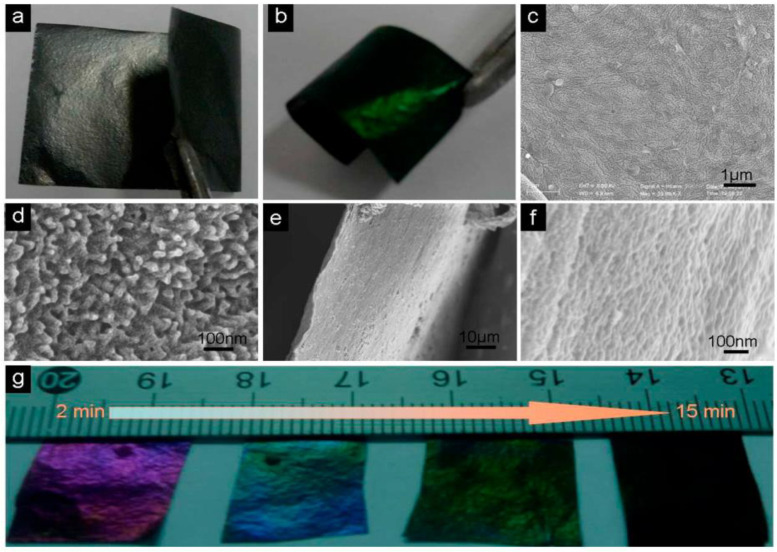
(**a**) Flexible graphene paper. (**b**) Graphene/PANI paper. (**c**,**d**) SEM micrographs of the surface of graphene/PANI paper. (**e**,**f**) SEM micrographs of cross sections of graphene/PANI paper. (**g**) Graphene/PANI papers with different electropolymerization times (From left to right: 2, 5, 10, 15 min). Reproduced from ref. [51], copyright 2013, Royal Society of Chemistry.

Although conducting polymers, such as polyaniline (PANI) and polypirrol (PPy), have outstanding properties, they alone might not be suitable as electrodes in devices such as supercapacitors. In order to enhance the electrochemical performance, they have been combined with graphene. A major advantage of using graphene as electrode is that both surfaces are readily accessible by the electrolyte. This nanomaterial has a theoretical specific capacitance of ~20 uF cm^−2^, corresponding to a specific capacitance of 550 F g^−1^ when the entire surface area is used [48]. However, due to its strong agglomerating tendency, expected values are not in polymeric nanocomposites. For instance, PANI/nitrogen-doped graphene nanocomposites were synthesized by in situ polymerization [49] and displayed good cycling stability with a specific capacitance of 480 F g^−1^. Others were prepared via chemical precipitation method, resulting in a specific capacitance in the range of 300–500 F g^−1^ [50]. A flexible PANI/nitrogen doped nanocomposite was synthesized via electropolymerizing of PANI nanorods onto nitrogen-doped graphene paper, which retained the original flexibility of graphene paper. The supercapacitor electrode showed very high specific capacitance (about 770 F g^−1^) and outstanding cycling stability attributed to the homogeneous growth of the polymer on graphene (Figure 10) [51], thus it is perfect candidate for application in the manufacture of transportable energy devices.

A self-standing 3D PANI/rGO foam was prepared via an in situ polymerization method with the aid of a template, to obtain a specific capacitance of 700 F g^−1^, which preserved around 90% of the original value after a hundred of cycles [52]. In addition, a PANI-grafted rGO nanocomposite electrode with fibrillar morphology has also been developed [53], which showed a high electrical conductivity at 25 °C and a specific capacitance of 250 F g^−1^.

**Figure 11:**
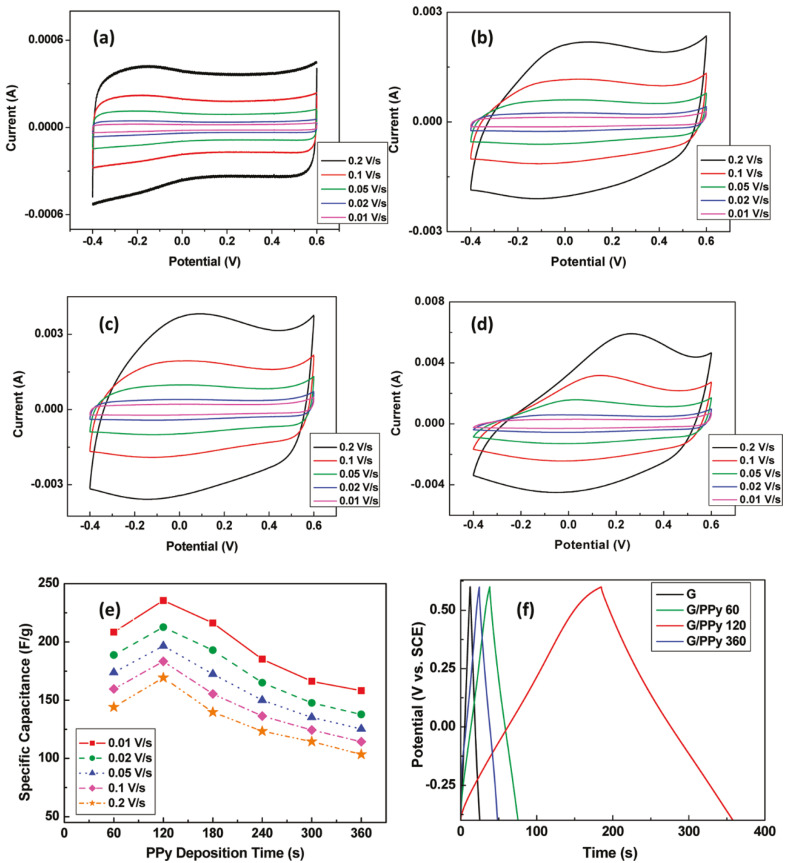
Cyclic voltammograms for (**a**) graphene, (**b**) graphene/PPy (60 s electrodeposition), (**c**) G/PPy (120 s electrodeposition), and (**d**) G/PPy (360 s electrodeposition) at different scan rates. (**e**) Specific capacitance of all G/PPy electrodes by electrodeposition time. (**f**) Galvanostatic charge–discharge curves for the electrodes in (**a**–**d**). Reproduced form ref. [55], copyright 2011, American Chemical Society.

Graphene/PPy composite fibers with diameters ranging from 15 to 80 μm have been developed via wet-spinning approach [54]. The fibers showed elevated conductivity and flexibility, thus providing substantial benefits as flexible, low dense electrodes for electrochemical supercapacitors. The complete supercapacitor with H_2_SO_4_–polyvinyl alcohol (PVA) electrolyte was prepared, which was converted into a textile for wearable electronics. Binder free composite electrodes with multilayer graphene and PPy nanowires have also been prepared, which showed a maximum capacitance of 160 F g^−1^ for the highest scan speed. Novel flexible graphene/PPy nanocomposite films were manufactured using a pulsed electropolymerization technique [55]. A maximum capacitance of 240 F g^−1^ was attained for a whole buildup time of only 2 min (Figure 11), about four-fold that of the raw graphene alone. Different scan rates were tested, from 0.01 to 0.2 V/s. This flexible supercapacitor showed exceptional energy (∼33 Wh/kg) and power density, ∼1200 W/kg, at the lowest scan speed. This enhancement was ascribed to the advantageous nucleation of polymeric segments at defect points of the graphene surface. On the other hand, nanoscale fillers, such as TiO_2_ have been mixed with graphene for the development of flexible capacitors. For instance, TiO_2_/graphene/PPy composites were synthesized in four stages [56]: first, TiO_2_ precursor and GO films were prepared by direct mixing and drying. Second, hydriodic acid (HI) was used as green reducing agent to obtain rGO/TiO_2_ films. Third, annealing was performed at different temperatures to obtain films with diverse titania crystalline phases. Lastly, PPy was deposited onto rGO/TiO_2_ composites. The TiO_2_ nanoparticles improve nanocomposite wettability, leading to high capacitance and good cycling stability. Moreover, different TiO phases had different behavior. Anatase had higher capacitance while rutile had better stability.

Phenolic resin-based laser-induced graphene patterns have also been prepared for application in flexible supercapacitors [57]. This type of nanocomposites, fabricated with a laser under ambient conditions, present very interesting properties, including 3D porous structures, low electrical resistance, and good mechanical performance. In addition, this approach shows numerous advantages including low cost, easiness, excellent film formation ability, as well as tunable structure and composition. More recently, polyacrylonitrile (PAN) nanofiber mats including GO with a core–shell microstructure were developed via coaxial electrospinning and hot-pressed into nanocomposite films [58]. The hot-pressing induced conformational changes in PAN, leading to the formation of an electroactive phase with high dielectric constant. Simultaneously, the GO was reduced into rGO. The resulting rGO/PAN composites showed thermally stable dielectric properties with a high dielectric constant over a broad temperature range. This work provides an effective approach for the development of flexible composite dielectric films for high-temperature electronic applications.

### 3.3. Graphene in Flexible Electronic Textiles

Wearable electronic devices, like e-textiles, are of great interest for use in multifunctional fabrics, portable electronic devices, and wearable displays [59]. To accomplish marketable demands, an e-textile needs to be light, stiff, conductive, flexible, and wearable. Carbon-based nanomaterials are suitable since they meet all of these conditions. In this regard, woven fabrics with graphene display outstanding elasticity and strain sensitivity.

**Figure 12:**
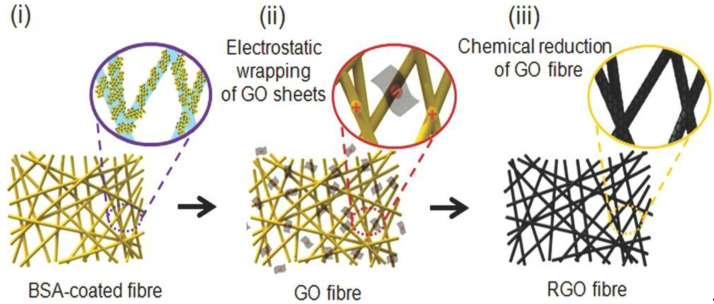
Schematic illustration of the stages used to prepare the nylon 6-rGO yarns: (i) functionalization with BSA; (ii) Electrostatic assembly; (iii) reduction of GO fibre. Reproduced from ref. [60], copyright 2013, Royal Society of Chemistry.

A novel e-textile that can be prepared as yarns or fabrics was prepared with rGO and nylon-6 [60]. They were obtained by electrostatically assembling GO with bovine serum albumin (BSA), a common adhesive for GO adhesion onto textiles. This technique can be applied to many current textiles, such as cotton, nylon, polyesters, and so forth. The composites exhibited an elevated electrical conductivity (>1000 S/m) that remained under successive washing cycles at different temperatures. The yarns were made-up in three stages (Figure 12): firstly, functionalization of electrospun nylon-6 yarns with BSA molecules (yellow dots) was carried out via simple dipping; secondly, an electrostatic self-assembly between the GO nanosheets and BSA-functionalised yarns was attained. Finally, rGO/nylon-6 yarns (black color) were prepared using HI as a reducing agent at low temperatures.

In a recent study, the production scale dyeing approach was used to prepare a fabric coated with GO, which was converted into rGO with a sustainable reductor to yield extremely conducting textile electrodes. Then, using the layer-by-layer method, these textiles were subsequently coated with conductive polymers to develop breathable, flexible, and washable electrodes [61]. The effect of post-treatment with ethylene glycol (EG) and dimethyl sulfoxide (DMSO) was also investigated, which further enhanced the electrical conductivity. These graphene-coated wearable electronic textiles can be used in health monitoring systems and biomedicine. In another work, a multifunctional wearable nanocomposite with graphene was developed via laser scribing technology [62]. A thin layer of polydimethylsiloxane was deposited regularly onto graphene-textile film, which improved abrasion resistance, and extended durability, whereas preserved flexibility. By controlling the voltage, constant temperature heating can be attained, thus enabling the detection of human movement and pulse signals.

### 3.4. Graphene in Flexible Transistors

Graphene is highly valuable for flexible electronics but simultaneously needs band-gap opening for digital applications. This nanomaterial can be applied as an electrode in field-effect transistors (FETs), which need high transparency combined with elevated conductivity. It can act as a source and drain, as well as a channel layer in any type of FET structure. The idea is to find a great capacitive, easily printable, tough, and well-matched material for gate dielectric. Polymeric electrolytes can be an optimum selection, though their time-consuming response with frequency impedes attaining a good TFT performance. Taking into account compatibility with graphene, GO can be chosen as an insulator, though the development of high-quality GO film is essential. In this regard, organic field-effect transistors (OFETs) are more attractive as they can easily be fabricated by printing method and exhibit a good on/off ratio.

**Figure 13:**
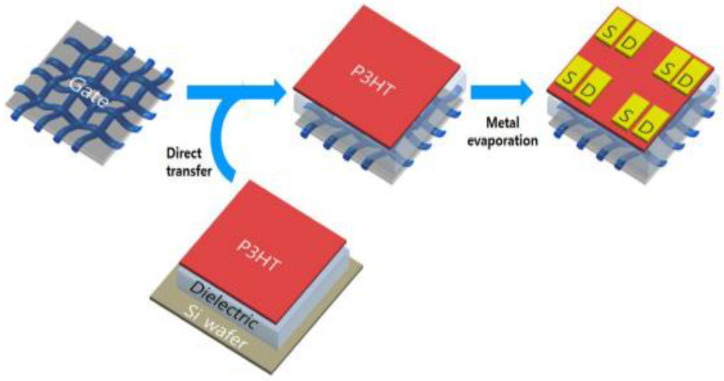
Representation of the fabrication process of the FET based on graphene/silver nanoparticle (AgNP) textile. P3HT: Poly(3-hexylthiophene); S: Source electrode; D: Drain electrode. Reproduced from ref. [63], copyright 2016, MDPI.

Highly conductive and flexible graphene-based textile composites for OFETs have been recently developed. They were arranged by vacuum filtration and wet-transfer of GO onto flexible PET textiles combined with the addition of AgNPs. In particular, the transistor devices were fabricated with a bottom-gate top-contact structure, as depicted in Figure 13. Poly(3-hexylthiophene) (P3HT) was selected as the solution-processable p-channel semiconductor [63]. A flexible poly(vinylidene fluoride-co-hexafluoropropylene) P(VDF-HFP) gel layer and an ionic liquid ((EMI)(TFSA)) were employed as a high capacitance gate dielectric and a mechanically tough transporter, respectively. Upon repeated spin-coating of the ion gel and the P3HT layers onto a clean silicon wafer, the two-fold layer was cut and moved onto the AgNP/graphene electrode placed on a PET textile.

Analogously, flexible fiber-type FET with graphene/Ag hybrid fibers as highly conductive electrodes were developed via wet-spinning and an adapted wet-drawing process. The fibers were then mixed with AgNPs, which showed elevated electrical conductivity, up to 16,000 S cm^−1^. The transistors presented excellent stability in terms of device performance, which was preserved following a thousand of bending cycles and preserved for 1 month out of the glove box [64].

## 4. Conclusions and Future Perspectives

Portable and wearable electronics are becoming extremely popular, since devices make life more secure, healthier, and more relaxed. In particular, flexible electronics have gone through important progresses in recent years, due to the miniaturization of technology and the wireless revolution. Henceforth, novel materials need to be explored. Graphene is regarded as one of the most talented materials for the next-generation of flexible electronic applications, due to its brilliant optical, mechanical, and other characteristics. However, most of the graphene-based devices developed up to date are time-consuming and need multi-stage fabrication routes, which are neither scalable nor suitable for industrial production. Moreover, they typically display poor electrical conductivity, washability, and flexibility. In order to solve these issues, they can be combined with polymers to manufacture nanocomposites with enhanced stretchability, superior mechanical strength, conductivity, and stability. Another approach is the combination with other nanoscale fillers like silver nanowires, that also display optimal properties for flexible electronic applications [65]. However, in order to use them in commercial applications, many challenges need to be addressed. For instance, novel approaches that permit to make high-quality graphene films with controlled size, composition, and electronic properties need to be established, since these characteristics determine the device performance. Regarding electrodes for OSCs, an optimum balance between conductivity and transparency is desirable. In addition, the real specific surface area of graphene nanomaterials is smaller than the forecasts due to their intense agglomeration tendency, and the mixture with polymers makes it even worse. Hence, novel synthetic approaches to avoid aggregation are required. Additionally, in order to synthesize composites with outstanding performance, the interfacial graphene–polymer interactions need to be studied. Novel doping or functionalization approaches that are well-matched with the fabrication process of flexible electronic devices need to be considered. Recently, laser driven integration of graphene into polymers has been reported as an effective approach for the development of hybrid structures with outstanding mechanical resistance, cyclability, and chemical stability for flexible electronic applications, including electrodes for energy storage, and electrochemical and bending sensors [66]. More importantly, economic means to synthesize graphene and its derivatives at a large scale are crucial. Though complications and challenges still exist, it is envisaged that, in the near future, scientists will be able to improve the performance by merging the good qualities of graphene and organic polymers to develop high-performance flexible electronic devices. The present and near future marketplace for polymer/graphene applications is determined by their manufacturing processes. Once each production path is well established, a wide-ranging practical implementation of these nanocomposites will be attained.
